# Acceptance and needs of medication literacy education among children by their caregivers: A multicenter study in mainland China

**DOI:** 10.3389/fphar.2022.963251

**Published:** 2022-09-13

**Authors:** Xiaolin Xu, Zhonglan Wang, Xialei Li, Ying Li, Yu Wang, Xuexin Wu, Lina Hao, Xiaoling Wang

**Affiliations:** ^1^ Department of Pharmacy, Beijing Children’s Hospital, National Center for Children’s Health, Capital Medical University, Beijing, China; ^2^ Department of Pharmacy, Children’s Hospital Affiliated to Shandong University, Jinan, China; ^3^ School of Pharmaceutical Sciences, Shandong University, Jinan, China

**Keywords:** caregivers, children, medication literacy education, popular science, health promotion, Lasswell’s communication mode

## Abstract

**Background:** This study aims to investigate the needs of child caregivers for popular science about safe medication for children, to deeply explore the characteristics of child caregivers’ demand for safe medication and the shortcomings of current popular science work, and then to seek better coping strategies to ensure children’s safe medication.

**Methods:** A questionnaire was designed based on Lasswell’s “5W” communication model to investigate the needs of child caregivers in terms of content, channels, and forms of popular healthcare science on the safe usage of children’s medication.

**Results:** The primary ways caregivers receive popular healthcare science education concerning safe medication usage knowledge are through medical institutions, notification by medical staff, and personal media. The caregivers of children have a high demand for the presentation of text, pictures, and videos in three forms of popular healthcare science content. Caregivers placed significant importance on the popularization of safe medication usage for children. The survey results showed that the top 3 ways for caregivers to think that the quality of popular healthcare science content was “very good” came from medical institutions, medical staff notifications, and personal media, effectively increasing popular healthcare information accuracy. The intelligibility and pertinence of content expression are urgently needed within the caregiver population.

**Conclusion:** Caregivers are very concerned about the popular science of safe medication for children, and are willing to learn about relevant content. Guided by the demand, we should actively disseminate accurate and easy-to-understand popular science about safe medication for children to caregivers through online or offline channels so as to promote safe medication for children.

## 1 Introduction

Health literacy has been recognized internationally as one of the important public health strategies in maintaining national health as well as achieving health equity (World Health Organization, 2016; Santos et al., 2017; Sentell et al., 2020). In 2018, the overall level of health literacy among Chinese residents was only 17.06%, which is still at an all time low (The Central People’s Government of the People’s Republic of China, 2018). Low levels of health literacy and inappropriate or inaccurate health communication may lead to medication errors within the public domain (Taylor and Harding, 2001). China’s“ Opinions of the State Council on Implementing Healthy China Initiative (2019–2030)” (State Council, 2019), contained one of the first major healthcare actions called the “Health Knowledge Popularization Action”, which mentions rational drug use as the skillset and healthcare information that citizens should be most familiar with learning, retaining, and implementing. Medication education is not only an important way for the public to obtain information, but also is an indispensable strategic policy to improve the health literacy of the people as a whole (Aslam et al., 2020).

According to the “2015 Children’s Medication Safety Report” released by Safe Kids Worldwide-China (Safe Kids Worldwide, 2015), drug poisoning was the leading cause of poisoning in children, and is still on the rise. A study in the United States (Schillie et al., 2009) showed that approximately 71, 224 children in the United States are admitted to the emergency department due to drug overdose each year. Therefore, these admissions account for 68.9% of the emergency department visits for children with poisoning, and the proportion of drug poisoning was twice that of non-drug poisoning. Among these cases of drug overdoses, 82.2% originated from self-medication administration without professional guidance, with medication errors and abuse accounting for 14.3% of the drug overdoses (Schillie et al., 2009). Medication education is one of the most important means of improving the health literacy of child caregivers in order for the caregivers to rationally administer drugs, thus, reducing the medication errors in children.

Due to the post-epidemic era, the public now has a heightened awareness of health, significantly improving an individual’s awareness and physical health status (Sentell et al., 2020). Under these circumstances the popularization of health science literature is a public service; therefore, it should pay close attention to public satisfaction and demands for such information. The public is a key element and the target audience of health science literature, thus the needs of the public are the initial starting point when disseminating health related information (Ren and Zhao, 2014; Centers for Disease Control and Prevention, 2019). Health science literature’s informatization is currently increasing; thus, the public’s needs for health-based information are also changing. Furthermore, there are differences in the public’s demand for drug science information among different groups and are affected by factors such as natural conditions, economic development, and personnel composition (Zhong et al., 2020).

The nature of the dissemination of medication knowledge is the dissemination of information, with the purpose of trying to influence the audience. The dissemination process of information includes: Who, Say What, In Which Channel, To Whom, With What Effect (Lasswell, 2013). Based on Lasswell’s 5W communication model (Lasswell, 2013), this study has designed a questionnaire to investigate child caregivers’ needs of popular science about safe medication for children. Furthermore, we have explored the characteristics of children’s caregivers’ demand for safe medication information as well as the shortcomings of the current popular science about safe medication. We then sought better countermeasures to enhance the dissemination of safe medication so as to improve the caregivers’ medication literacy and reduce the incidence of children’s medication errors. The results of the questionnaire survey are reported as follows.

## 2 Materials and methods

### 2.1 Study design

In this study from 1 April 2021 to 31 September 2021, the sample was selected using two-stage stratified sampling. In the first stage, we selected five children’s hospitals from cities at prefecture level in Central China, North China, South China, Northwest China, and Northeast China. In the second stage, The sample size of the questionnaire was calculated by the proportional probability sampling method (PPS). According to the information standard of the information system of hospitalized children in the previous year in each hospital, 10% of the total number was selected. Caregivers of hospitalized children aged 0–6 years were randomly selected from each hospital. Face-to-face interviews were conducted by trained interviewers with the primary caregivers of children selected. Primary caregivers were defined as those who regularly cared for the children, such as parents or grandparents.

The inclusion criteria include: 1) The subjects are long-term and stable caregivers of the children; 2) The children in the family are taking medicine or have a history of taking medicine; and 3) The doctor has confirmed that they have good communication and understanding skills. The exclusion criteria include: 1) Eliminate repeat questionnaires; 2) Questionnaires with inconsistent logic in the front and back options; 3) Filling time < 200 s.

#### 2.1.1 Design of the questionnaire

Based on Lasswell’s “5W” communication model (Lasswell, 2013), namely who (communication subject), say what (communication content), to whom (communication object), by which channel (communication channel), and what effect (communication effect), a questionnaire was designed to investigate the needs of a child’s caregiver in terms of content, channels, and forms of popular health related literature.

#### 2.1.2 Questionnaire evaluation criteria

Using the Likert 5-level scoring method we assigned points ranging from 1 to 5 and correspond to 1 “no need at all”, 2 “not very necessary”, 3 “general need”, 4 “more need”, and 5 “very necessary”. Single-choice questions such as demographic and sociological information, including gender, permanent residence, highest education level, monthly household income, ethnicity and other basic information, are filled out on the form by the volunteer. Answers to the multiple-choice questions are guided in the presence of a pharmacist. When the selected answer conforms to the child’s actual situation, the answers are considered valid.

#### 2.1.3 Questionnaire quality control

The questionnaire quality control primarily includes investigation and data controls. 1) Prior to the investigation, we formulated an investigation implementation plan, and conducted a unified training for investigators and quality control personnel. If the respondents do not understand the question, they can ask the investigator; however, the investigator is not allowed to answer the relevant content of the question that involves the answers of the questionnaire as well as does not give leading questions or prompts. 2) During the data entry stage the double entry of questionnaire data is implemented, and a consistency check is carried out to ensure the entered data’s accuracy.

### 2.2 Statistical analysis

The data was imported into the SPSS 25.0 software for analysis. The count data are expressed as frequencies and percentages. Univariate analysis compared the importance of safe medication and the demand for popular health information among caregivers with different demographic and sociological characteristics. The reliability and validity of the questionnaire were evaluated *via* the retest reliability and content validity index, respectively.

## 3 Results

### 3.1 Questionnaire distribution, reliability, and validity evaluation

A total of 1,002 questionnaires were distributed in this survey. According to the exclusion criteria, invalid questionnaires such as incomplete and irregular filling were screened out, and 963 valid questionnaires were recovered, with an effective rate of 96.1%.

Five clinical pharmacists from different regions (2 chief pharmacists and 3 deputy chief pharmacists) evaluated and revised the questionnaire’s semantics, expression habits, and professional nature. The content validity index of experts evaluation was found to be 0.928. After the questionnaire was formulated, 80 parents were pre-investigated. 1 week later, 43 parents were selected to repeat the same questionnaire, and the test-retest reliability was 0.767.

### 3.2 The main body of dissemination of popular health related information on safe medication use for children

**FIGURE 1:**
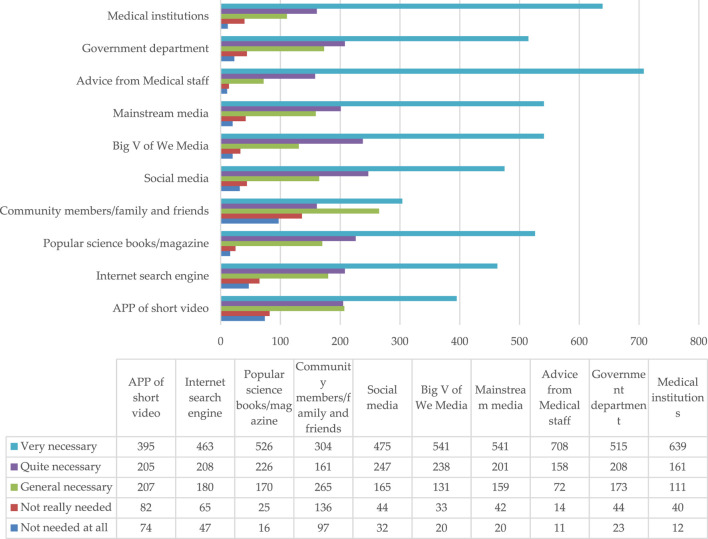
The needs of caregivers based on the supply channels of popular health related information regarding children’s safe medication usage knowledge.

The questionnaire survey results (Figure 1) show that the medical structure, medical staff, mainstream media, self-media big V, and popular science books/magazines were the main propagators of health related information on safe medication use for children. The results showed that the public had a higher demand and trust for health information popularizing medication use by medical institutions and personnel.

### 3.3 Demands of child caregivers for popular health related information concerning the safe medication usage among children

**FIGURE 2:**
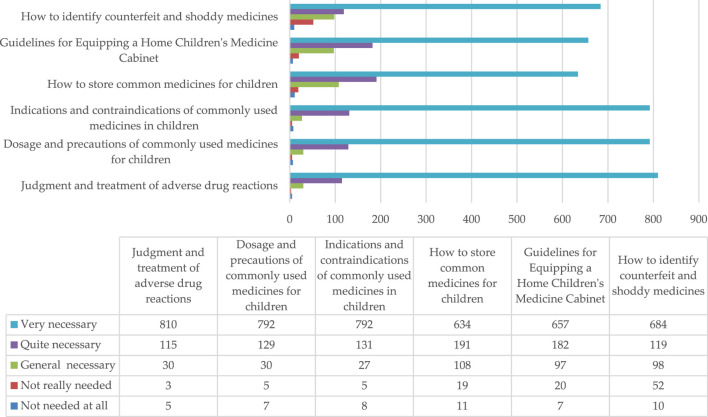
Caregivers’ need for scientific content on safe medication usage for children.

The survey results show that (Figure 2), the caregivers have an increased demand for information regarding the indications, contraindications, usage, dosage, precautions, and the diagnosis and treatment of adverse reactions of commonly used medication for children. Moreover, the demand for knowledge is used to identify counterfeit and sub-par drugs, equipping households with children’s medicine boxes, as well as the storage methods of children’s medication. This showed that the caregiver group had an excellent grasp of the basic information on preventing misuse; however, the understanding of children’s commonly used drugs and the diagnosis and treatment of adverse drug reactions was limited. Therefore, caregivers urgently needed targeted healthcare information popularization and education on medication and its usage.

### 3.4 Recipients of popular health related information regarding safe medication usage among children

In this study, the children’s caregivers were the recipients of popular health related information regarding the safe medication usage for children. Among the children caregivers surveyed, female respondents accounted for 82.5% of the total sample size, and male respondents accounted for 17.5%; in terms of age, the proportion of respondents aged 31 to 40 was 54.9%; in terms of permanent residence, the majority of respondents were urban residents, accounting for 89.9%; and in terms of education, 66.3% of respondents had a bachelor’s degree or higher (Table 1).

**TABLE 1:** Sociodemographic characteristics of the children’s caregivers (*n* = 963).

Variable	*n*	%
children’s caregivers	963	—
Gender		
Male	169	17.5
Female	794	82.5
Age		
25 years and under	50	5.2
26 ∼ 30 years old	209	21.7
31∼ 40 years old	529	54.9
41 ∼ 50 years old	127	13.2
51 years and over	48	5.0
Permanent residence		
Urban	866	89.9
Rural	97	10.1
Highest education		
Junior high school and below	54	5.6
High school	46	4.8
Technical secondary school	45	4.7
Junior college	180	18.6
Undergraduate	527	54.8
Postgraduate	111	11.5
Marital status		
Married	936	97.2
Divorced	20	2.1
Widowed	7	0.7
Average monthly income per person in family		
Below 2000 yuan	59	6.1
2000∼4,000 yuan	186	19.3
4,000∼6,000 yuan	267	27.8
6,000∼8,000 yuan	169	17.5
More than 8,000 yuan	282	29.3
Current/pre-retirement industry		
Office staff and related personnel	404	42
Heads of state organs/party group organizations/enterprises/institutions	73	7.6
Soldier	1	0.1
Production personnel in agriculture/forestry/animal husbandry/fishery/water conservancy	13	1.3
Other employed/unemployed	270	28.0
Business/service personnel	82	8.5
Operators and relevant personnel of production/transportation equipment	18	1.8
Specialized technical staff (excluding medical staff)	101	10.5
Students	1	0.1

### 3.5 Ways and media forms for child caregivers to accept popular health related information on children’s safe medication usage

#### 3.5.1 Approaches to receiving popular health care information on the safe medication usage for children

**FIGURE 3:**
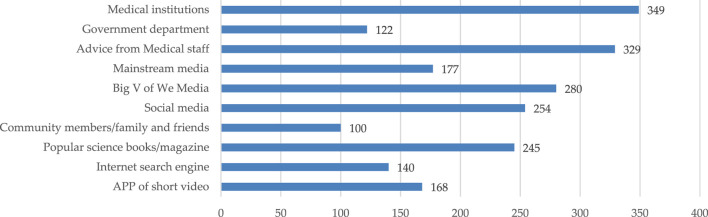
Access to healthcare science education regarding the safe usage of children’s medication.

The results of the survey (Figure 3) show that the primary ways for caregivers to receive popular health care education regarding knowledge on children’s safe medication usage were provided by medical institutions (publicity boards and pamphlets), medical staff notifications, and big V of We Media (Dr. Clove and Dr. Chunyu). Furthermore, unique groups such as medical institutions and personnel used their highly specialized knowledge and healthcare skillsets to conduct popular health care education topics and classes for the caregivers. In addition, social media also played an important role in disseminating health science information (Edington et al., 2016; Stellefson et al., 2020). Therefore, with the development of internet technology, the big V of We Media gained a significant audience base. In comparison, more traditional communication methods such as mainstream media (central or local TV stations and radio programs) and popular health care related science books and magazines are needed to further strengthen the knowledge and publicity concerning the safe usage of children’s medication.

#### 3.5.2 Accepting the media carrying form of popular science on safe medication for children

**FIGURE 4:**
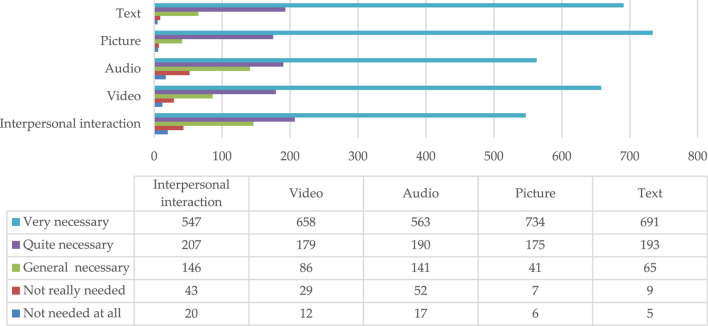
Demand from caregivers of a scientific form of healthcare knowledge concerning the safe usage of children’s medications.

The survey results (Figure 4) show that the children’s caregivers had an increased demand for three forms of popular health care content: text, picture and video.

### 3.6 The effect and satisfaction of children’s caregivers receiving popular health care related science on safe medication for children

#### 3.6.1 The degree of attention paid by children caregivers concerning the popularization of the safe usage of children’s medication

The results of the survey showed that the children’s caregivers paid more attention to the popularization of children’s safe medication usage, with a large percentage of caregivers (99%) attributing great significance to this particular set of knowledge. This shows that the children’s caregivers believed that it was an excellent questionnaire needed to understand the safe usage of children’s medication through health care related science popularization which could, reduce medication errors and the risk of unreasonable medication administration. However, nearly half of the respondents (47.6%) indicated that they had never received popular health care education regarding the safe usage of children’s medication. This indicates the educational gaps and deficiencies within the field of health care information popularization as well as the education on the safe usage of children’s medication; thus, showing a mismatch between supply and demand.

#### 3.6.2 Evaluation of the quality of popular science content obtained by different dissemination channels

**FIGURE 5:**
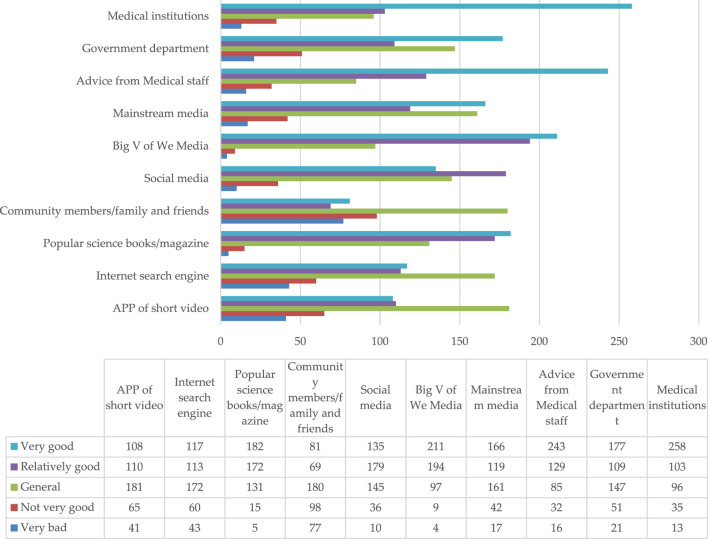
Overall quality of knowledge regarding the safe usage of children’s medication obtained from various sources.

The results (Figure 5) also showed that among all the channels that can disseminate popular health care information on the safe usage of children’s medication, the top three channels that considered the quality of popular health care content to be “very good” were medical institutions, medical staff notification, and the big V of We Media. This ranking was similar to the rankings mentioned above for the primary ways that the caregiver group receives popular healthcare education, thus, proving the outstanding contributions of medical institutions, medical personnel, and We Media influencers on the popularization of the information surrounding the safe usage of children’s medication. The caregiver group affirmed the quality of the information transmitted through the above channels. Conversely, the quality of information obtained from the community resident, search engines, and the APP short video channels showed a bad or very bad situational outcome.

#### 3.6.3 Suggestions for improving the overall quality of the existing popular healthcare information content

**FIGURE 6:**
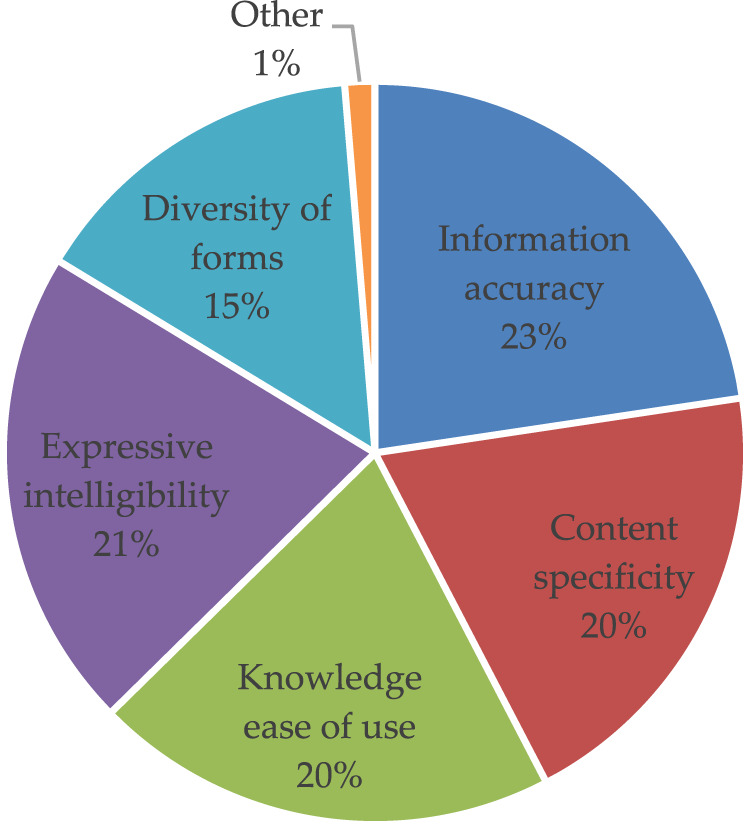
Specific aspects of improving the overall quality of science content.

The results (Figure 6) show an urgent need for the caregiver group to effectively increase the accuracy of popular healthcare information as well as the intelligibility and pertinence of the content expression. Furthermore, some subjects pointed out that it was crucial to focus on the timeliness of the content of popular healthcare education. Drug and drug information is updated quickly over time, outdating some of the information found in hospital propaganda columns. In addition, in terms of content, it is necessary to strengthen and improve the construction of the healthcare information popularization system required for the safe usage of medications used to treat rare diseases in children.

## 4 Discussion

The respondents believed that they urgently needed popular healthcare education; however, they had very limited access to popular healthcare information on safe medication usage. The data shows that the popular healthcare work in this field of safe medication usage should be vigorously strengthened. The ways caregivers have received popular healthcare education have proven that the contributions made by medical institutions and medical staff in popularizing knowledge about the safe use of medication for children as well as the high recognition of the quality of the information disseminated are outstanding. Conversely, the quality of information obtained from community residents, search engines and APPs of short video channels has shown a poor or very bad situation; thus, it reflected a social phenomenon that proves dissemination of information from the network environment was mixed, and contained many untrue rumors (Berland et al., 2001; Kunst et al., 2002; Mheidly and Fares, 2020). It was suggested that relevant departments and platforms still needed to pay more attention to the quality of popular healthcare information.

The problems that exist within the quality of the current popular healthcare information have hindered the further improvement of health literacy, including the information expression is not authentic and reliable as well as the information content is not updated in a timely manner. Currently, the contradiction between the public’s urgent need for healthcare science popularization and the low degree of trust in content from some of the dissemination channels has been an urgent and significant problem that needs to be solved for the development of healthcare popularization works. Comparing the existing communication channels shown in Figure 2, it has been revealed that the mainstream media has not currently exerted significant, influential power. Therefore, it is imperative to make full use of mainstream media resources (Robinson et al., 2014) in order to disseminate large amounts of information regarding healthcare content with high fidelity and to the desired audience in a timely and rapid manner. The primary age group of people who are the children’s caregivers is the elderly. Unfortunately, this age group has a fixed way of thinking and uses traditionally passed down remedies that may or may not have any medical evidence of working. It is the belief of the younger generation to be disseminators of peer-reviewed evidence-based medical advice and knowledge to this group of caregivers in order to better provide care to the children and circumvent any adverse medication related reactions.

With the development of economy, society, and network technologies, China’s popular healthcare related work has shown an informatization trend; thus, the needs of the caregivers as well as the popular healthcare information have become more diversified. Designing and carrying out popular healthcare related work in accordance with the “demand-oriented” principle could effectively improve the efficiency and satisfaction of popular healthcare works (Zhang and Ran, 2019; (von Rüden et al., 2021). Therefore, based on the results of this survey, the country and the government should place significant importance on the safe usage of children’s medication, and organically combine the formulation of laws and policies suitable for the country’s national conditions and effectively implement the existing guidelines to further promote the “Popularization Action of Children’s Safe Medication” at the macro level. For medical institutions and practitioners related to popular healthcare information, the accuracy and pertinence of popular medical information should be strictly controlled with professional and obscure healthcare knowledge being converted into layman’s easy-to-understand language and presented so that the caregivers can easily grasp and readily use. Moreover, in the process of carrying out the relevant publicity and education of popular healthcare information, special attention should be paid to disseminating different and personalized methods of education for different educational levels, income levels and population distribution regions (Ruben, 2016; Lin and Huang, 2018). Electronic media such as media platforms and search engines should rely on the strong development of network technologies as well as scientific management in order to improve the authoritative, professional, and comprehensive medical knowledge database. This would improve the network environment, and meet the needs of caregivers regarding the knowledge needed for the safe usage of children’s medication (Morahan-Martin, 2004).

Popular science about safe medication is a word with Chinese characteristics. It is generally called medication consultation and medication education in other countries. Few studies have explored the needs of child caregivers for safe medication education in other countries. A study has been conducted in the United States to explore how children with chronic diseases and their parents want to learn about medicines and their perceptions on medication counseling provided by community pharmacists (Abraham et al., 2017). This is similar to our study in that the public’s demand for medication knowledge has been investigated. And the difference is that our research object is limited to child caregivers, while the above study includes children and caregivers. This inspires us, and we can also include children as subjects in the follow-up research to discuss children’s needs for popular science about safe medication.

There are a few limitations of this study including the proportion of males and females, age level, highest educational level, occupational status, and structure of the survey samples were significantly biased, which affected the data analysis results to a certain extent.

## 5 Conclusion

With the enhancement of health awareness, child caregivers pay more and more attention to the knowledge of children’s safe medication, and expect to learn as much as possible about relevant popular science works. Then, in response to the needs of caregivers, we should think about how to transmit effective and high-quality popular science works on safe medication for children.

From the results, we can see that, the use of big data and other information tools helps to realize the accuracy of popular healthcare content, and accelerate the construction process of popular healthcare science channels; therefore, improving the trust of information channels, the expression forms of popular healthcare science related information as well as the effectiveness of healthcare science popularization. Additionally, the content of safe medication for children that caregivers are most interested in should be focused on, analyzed, and tracked. Furthermore, through the use of content mining and user management technologies, we can integrate popular healthcare information and user data, observe the potential relationship between content characteristics and user behavior, and use big data analysis to understand child caregivers’ different needs for popular science about safe medication for children (Wang et al., 2017; Zhang and Ran, 2019). Professionals such as doctors and pharmacists need to ensure that the popular science of safe medication for children are scientific and easy-to-understand, and actively popularize the knowledge about children’s safe medication online (new media) or offline (face-to-face education) to improve the awareness of safe medication among caregivers for reducing medication errors of children at home (Hart et al., 2017).

## Data Availability

The raw data supporting the conclusion of this article will be made available by the authors, without undue reservation.
